# Reassessing the unifying hypothesis for hypercontractility caused by myosin mutations in hypertrophic cardiomyopathy

**DOI:** 10.1038/s44318-024-00199-x

**Published:** 2024-08-27

**Authors:** James A Spudich, Neha Nandwani, Julien Robert-Paganin, Anne Houdusse, Kathleen M Ruppel

**Affiliations:** 1 Department of Biochemistry, Stanford University School of Medicine, Stanford, CA 94305 USA; 2 Structural Motility, Institut Curie, Paris Université Sciences et Lettres, Sorbonne Université, CNRS UMR144, F-75005 Paris, France; 3 Department of Pediatrics, Stanford University School of Medicine, Stanford, CA 94305 USA

**Keywords:** β-Cardiac Myosin, Hypertrophic Cardiomyopathy, Interacting Heads Motif, Super-relaxed State, Unifying Hypothesis, Musculoskeletal System, Structural Biology

## Abstract

Significant advances in structural and biochemical research validate the 9-year-old hypothesis that cardiac hypercontractility seen in patients with hypertrophic cardiomyopathy is primarily caused by sarcomeric mutations that increase the number of myosin molecules available for actin interaction.

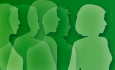

## Teaser

Human β-cardiac myosin exists in an ON-state where both myosin heads are accessible for interaction with actin, and an OFF-state where the heads are folded back onto their own coiled-coil tail, interacting with each other via an interacting-heads motif (IHM). Hypertrophic cardiomyopathy (HCM) mutations in β-cardiac myosin cause hypercontractility of the heart. Nine years ago, a unifying hypothesis proposed that hypercontractility caused by myosin HCM-associated mutations is primarily due to an increase in the number of ON-state myosin molecules, rather than altered fundamental alterations of functional myosin parameters such as intrinsic motor force, its velocity of movement along actin, or its ATPase turnover rate, all of which impact power output. We here revisit this unifying hypothesis in light of accumulated data measuring all these parameters, and the recent availability of a 3.6 Å-resolution structure of the human β-cardiac myosin IHM. Biochemical measurements show that nearly all myosin HCM mutations examined exhibit more ON-state myosin regardless of where they occur in the myosin head domain, consistent with the unifying hypothesis.

## Introduction

Hypertrophic cardiomyopathy (HCM) is the most prevalent inherited cardiac disease, affecting between 1 in 500 to 1 in 200 individuals (Semsarian et al, 2015; Virani et al, 2020), and a common cause of arrhythmias and heart failure. HCM patients present with left ventricular hypertrophy without predisposing conditions, which can progress to decreased left ventricular chamber volume and aortic outflow tract obstruction during heart contraction (systole). HCM is associated with mutations in genes encoding various muscle structural (sarcomeric) proteins (Seidman and Seidman, 2001), however, most mutations occur in either *MYH7* or *MYBPC3*, encoding β-cardiac myosin heavy chain and myosin-binding protein-C (MyBP-C), respectively. Mutations in these two genes account for over 80% of HCM patients with an identified genetic variant, with about 40% of those occurring in *MYH7* and 60% in *MYBPC3* (Alfares et al, 2015). The mutations in *MYBPC3* primarily cause protein truncations, leading to haploinsufficiency (Marston et al, 2009; van Dijk et al, 2009; Harris et al, 2011). The conventional view was that HCM mutations resulted in hyperdynamic cardiovascular physiology (hypercontractility) that is often seen as a supranormal ejection fraction (EF), a measure of contraction efficiency by echocardiogram, even before hypertrophy is manifest (Ho et al, 2002, 2009; Captur et al, 2014; Haland et al, 2016), and therapies have been aimed toward controlling the hyperactive physiology.

To understand the molecular basis of this mutation-induced hypercontractility, it is essential to reconstitute the functions of interest from purified proteins (Kawana et al, 2022). In this regard, a major advance in the field was the establishment of a mammalian myosin expression system in the mouse myogenic cell line C2C12 (Wang et al, 2003; Srikakulam and Winkelmann, 2004; Liu, Srikakulam and Winkelmann, 2008; Resnicow et al, 2010; Deacon et al, 2012). In collaboration with the Leinwand laboratory at the University of Colorado at Boulder, our lab at Stanford expressed and purified human β-cardiac myosin containing the relevant human ventricular essential (ELC) and regulatory (RLC) light chains. The availability of a pure human β-cardiac myosin molecule reconstituted with the relevant ventricular isoforms of light chains allowed investigation of the effects of HCM mutations on the contractile properties at the molecular level that might contribute to the hypercontractile state.

The prevailing view at the time was that HCM-causing mutations in the human β-cardiac myosin gene *MYH7* caused cardiac hypercontractility by increasing one or more of the fundamental parameters of the myosin, which are the velocity of contraction (v) and the force (F) the heart produces, since the power output (P) of the heart is P = F*v (Sivaramakrishnan et al, 2009; Moore et al, 2012). Two key factors affecting the ensemble force (F_ensemble_) of the heart are the intrinsic force (F_intrinsic_) of each myosin molecule, and the rate of the actin-activated myosin ATPase (k_cat_ = 1/t_c_, where t_c_ is the ATPase cycle time). Thus, F_ensemble_ = F_intrinsic_(t_s_/t_c_)N_a_, where t_s_ is the time a myosin head is strongly bound to actin in one chemomechanical cycle, t_s_/t_c_ is the duty ratio or the fraction of heads bound and producing force at a particular time during contraction, and N_a_ is the total number myosin heads in the muscle accessible for actin interaction.

The first measurements with two HCM mutant forms of purified human β-cardiac myosin, R403Q and R453C, appeared to fit this concept. Compared with wild-type, R403Q showed a 25% increase in actin-activated ATPase activity and a 15% increase in velocity (Nag et al, 2015), measured by in vitro motility assay, while R453C showed a 50% increase in F_intrinsic_ (Sommese et al, 2013), as measured with a dual beam laser trap. However, as seen in Table 1 (blue columns), filling in the matrix of measurements of ATPase, F_intrinsic_, and velocity for these two mutants as well as others indicated that while one parameter increased for a particular mutation, other parameters for that mutation often decreased, making it difficult to see how the ensemble of effects could lead to hypercontractility (Sommese et al, 2013; Nag et al, 2015). Furthermore, R663H and G741R mutants were not different from wild-type human β-cardiac myosin in any of the three parameters measured (Kawana et al, 2017; Sarkar et al, 2020).

**Table 1:**
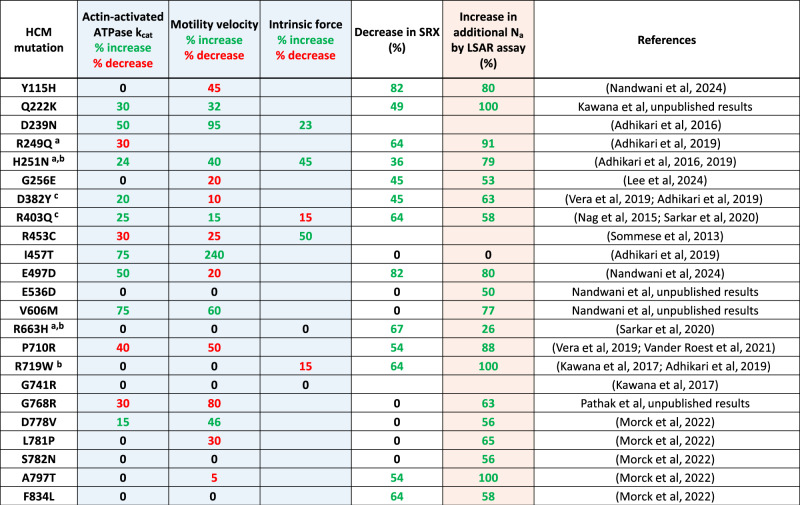
Summary of data available for 23 HCM mutant forms of human β-cardiac myosin.

Table showing data collected on important parameters of myosin function and regulation. The three blue columns are measurements of three fundamental parameters that determine power output from the muscle, where green values contribute to hypercontractility, red values contribute to hypocontractility, and black zeros indicate no change. The white column shows the percent decrease in SRX, setting the baseline for wild-type myosin at 55%. Thus, the % decrease SRX = (55−x)/55, where x = the measured SRX level for a particular HCM mutant myosin. The green values are generally assumed to indicate more myosin heads in play leading to hypercontractility, and black zeros indicate no change. The pink column shows the percentage increase in the additional number of myosin heads made accessible for interaction with actin (additional N_a_), setting the baseline of 0.57 as 0% additional heads released. The numbers derive from the formula percent additional N_a_ increase = (x−0.57)/0.43, where x = the measured long-tail short-tail ATPase ratio (LSAR) for a particular HCM mutant myosin and 0.57 is the average LSAR value for wild-type myosin. Thus, if the mutant protein gives the same LSAR value as the wild-type protein of 0.57, then 0% of the myosin heads are released by the mutation. If the mutant protein gives an LSAR value of 1.0, then 100% of the myosin heads are released by the mutation. The 6 residues that lie at the protein–protein interfaces of the IHM OFF-state described later in the text are indicated by symbols.

^a^Blocked head mesa residue, head-proximal tail interaction site.

^b^Free head residue, head–head interaction site.

^c^Blocked head residue, head–head interaction site.

**Figure 1:**
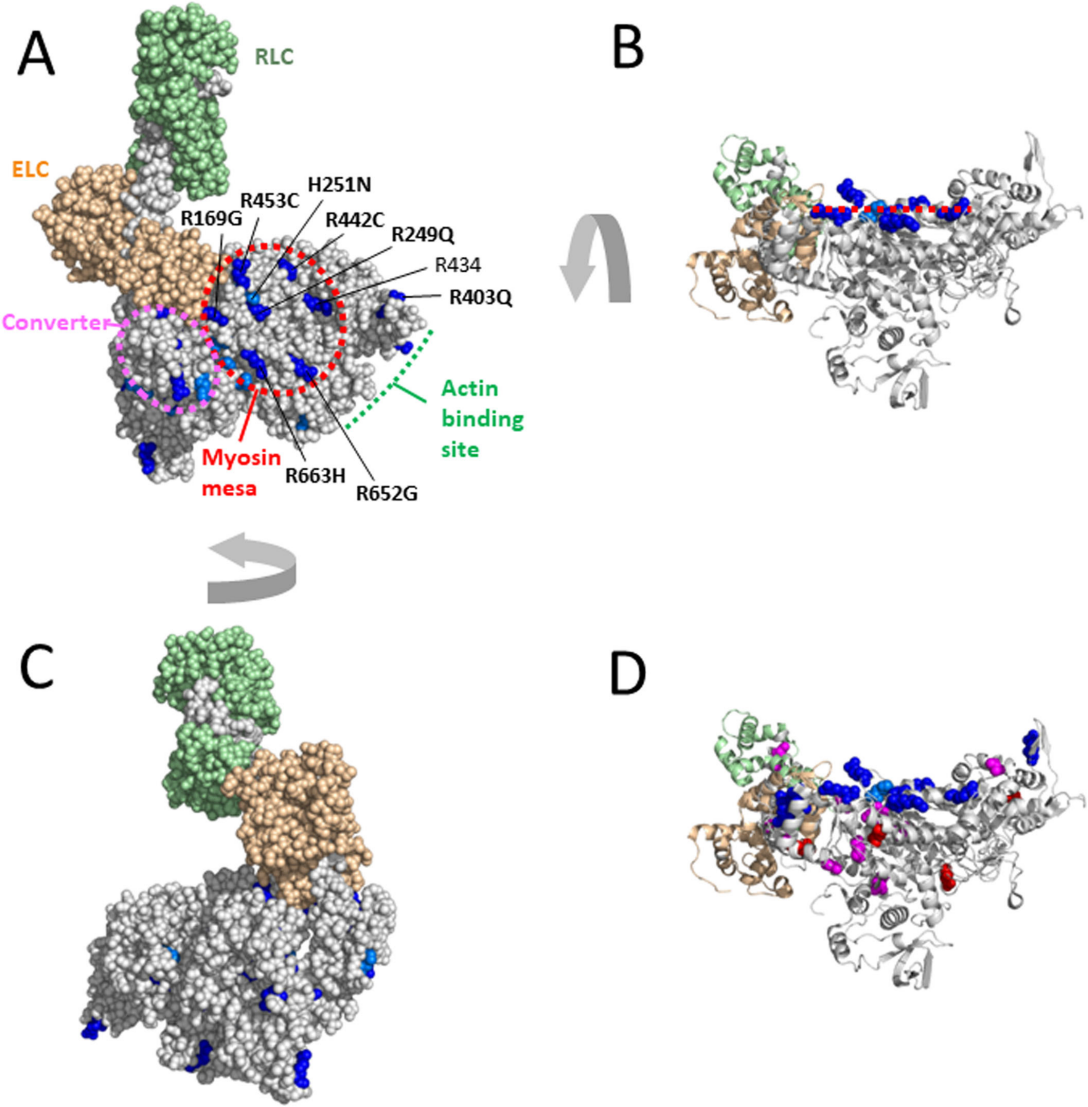
The myosin mesa and hypertrophic cardiomyopathy mutations. (**A**) The blocked head of the high-resolution human β-cardiac myosin OFF state (PDB 8ACT) with all arginine and histidine residues shown in dark and light blue, respectively. In this top view of the flat myosin mesa (red dashed oval), 6 arginine-based HCM residues and a single non-HCM-producing arginine, arginine 434, are shown, as well as a single histidine-based HCM residue, H251. The position of the myosin converter domain is illustrated by the purple oval. The myosin heavy chain is in light gray, the essential light chain (ELC) in light brown, and the regulatory light chain (RLC) in light green. (**B**) The molecule shown in (**A**) rotated ~90° to reveal the flatness of the mesa (red dashed line) and the positions of the mesa arginine- and histidine-based HCM residues (**C**) The opposite face of the molecule shown in (**A**). (**D**) The molecule shown in (**B**) but with the positions of all the HCM mutations from Table 1 shown. Negatively-charged and non-charged residues are shown as red and magenta spheres, respectively.

These results were unexpected and perplexing and one night in late 2014, after reading a book called “The Haunted Mesa,” a murder mystery by Louis L’Amour based in the Southwest where mountains with flat top surfaces called mesas dominate the landscape, a dream pointed to myosin having a mesa (see https://www.ibiology.org/cell-biology/muscle-biology/#part-4). Indeed, the crystal structures of myosins showed this relatively flat surface, but its importance was not appreciated. The next morning, analysis of the conservation of the residues on the myosin mesa surface across cardiac myosin species from mouse to man showed an unusual amount of conservation, suggesting there was something important about this surface (Spudich, 2015). Furthermore, when viewed from the correct angle, the majority of the HCM mutations we were studying at the time lay on the myosin mesa (Fig. 1A,B). In addition, most of those mutations resulted in positively-charged arginine residues being replaced by non-charged residues (Fig. 1A, bold residue numbers). It was therefore proposed that a sarcomeric protein with an overall negatively charged domain could normally interact with the myosin mesa of some myosin molecules in the sarcomere, keeping them in an OFF state by interfering with their ability to interact with actin (Spudich, 2015). Such heads could be being held in reserve and released by appropriate signaling mechanisms when higher power output from the heart was needed. A unifying hypothesis for hypercontractility caused by myosin HCM mutations was proposed: that most, if not all, myosin HCM mutations weaken the protein–protein interactions in this OFF state, increasing the number of heads accessible for interacting with actin (N_a_) and thereby causing the hypercontractility seen in HCM patients (Spudich, 2015). It was also proposed that MyBP-C might interact with the mesa and that dysregulation of MyBP-C could also lead to changes in N_a_ (Spudich, 2015), accounting for why the vast majority of HCM mutations occur in the two sarcomere proteins, β-cardiac myosin and MyBP-C. The status of this unifying hypothesis is evaluated in this editorial.

## The mesa surface and its role in the stabilization of the sequestered OFF-state

The unusual aspects of the myosin mesa surface compared to other surfaces on the myosin head domain are illustrated in Fig. 1A–C. In Fig. 1A & C, the arginine and histidine residues, both positively charged under physiological conditions, in the entire globular head domain of the myosin molecule are highlighted in blue. Notably, 6 of the 7 arginine residues at the mesa surface are known to cause HCM when mutated to a non-charged residue (Alfares et al, 2015). It will be interesting to see whether the one exception, arginine 434, is identified as an HCM mutation site in the future. Indeed, there is one report of an HCM patient who was screened for mutations in eight sarcomeric genes and this patient had only an R434T mutation (Wang et al, 2014), however, no additional reports are found in ClinVar to date. The single positively-charged histidine residue, H251, on the mesa surface also causes HCM when mutated. In addition, the HCM residue R403Q seen on the far right of Fig. 1A is readily visible from this view of the myosin head domain. Thus, this is a highly HCM-rich surface of the myosin molecule (Spudich, 2015; Homburger et al, 2016). In contrast, rotating the molecule shown in Fig. 1A to view the other side (Fig. 1C) reveals a more corrugated surface which has no surface arginine or histidine residues.

## The myosin proximal tail domain is involved in a sequestered OFF-state known as the Interacting Heads Motif (IHM)

The coiled-coil tail of human β-cardiac myosin is divided into two domains, subfragment 2 (S2), which follows directly after the subfragment 1 (S1) globular heads, and light meromyosin (LMM), which extends to the C-terminus of the molecule. LMM self assembles and forms the shaft of the muscle thick filament, while S2 is free to move away from the thick filament and bring the myosin heads in contact with the actin filaments. We define the proximal part of S2 (proximal S2) as the first ~15 heptads of the coiled-coil.

Another important protein–protein interaction, that of the proximal S2 of the myosin with its own globular S1 domain, derives from groundbreaking studies by Susan Lowey and Kathy Trybus (Trybus et al, 1982; Trybus and Lowey, 1984; Lowey and Trybus, 1995; Trybus et al, 1997; Lowey and Trybus, 2010) and then expanded by the laboratories of Ken Taylor (Wendt et al, 1999, 2001; Liu et al, 2003, 2006; Tama et al, 2005; Rahmani et al, 2021; Chen et al, 2024), Roger Craig (Woodhead et al, 2005; Jung et al, 2008b; Zoghbi et al, 2008; Zhao et al, 2009; Yang et al, 2020), Raul Padron (Alamo et al, 2008; Sulbarán et al, 2015; Alamo et al, 2018; Dutta et al, 2023), Matthias Gautel (Blankenfeldt et al, 2006), Stefan Raunser (Tamborrini et al, 2023), and others (Offer and Knight, 1996; Burgess et al, 2007; Jung et al, 2008a, 2011; Al-Khayat et al, 2013; Scarff et al, 2020; Heissler et al, 2021). Thus, a relevant protein interaction with the myosin mesa could be the proximal tail of the very same myosin molecule causing the two heads of the myosin to fold back onto its own tail in a structural OFF state that has been termed the interacting heads motif (IHM) (Wendt et al, 2001; Woodhead et al, 2005; Burgess et al, 2007; Al-Khayat et al, 2013; Woodhead et al, 2013; Lee et al, 2018; Craig and Padrón, 2022).

## The high-resolution structure of the human β-cardiac myosin IHM state supports the unifying hypothesis

**Figure 2:**
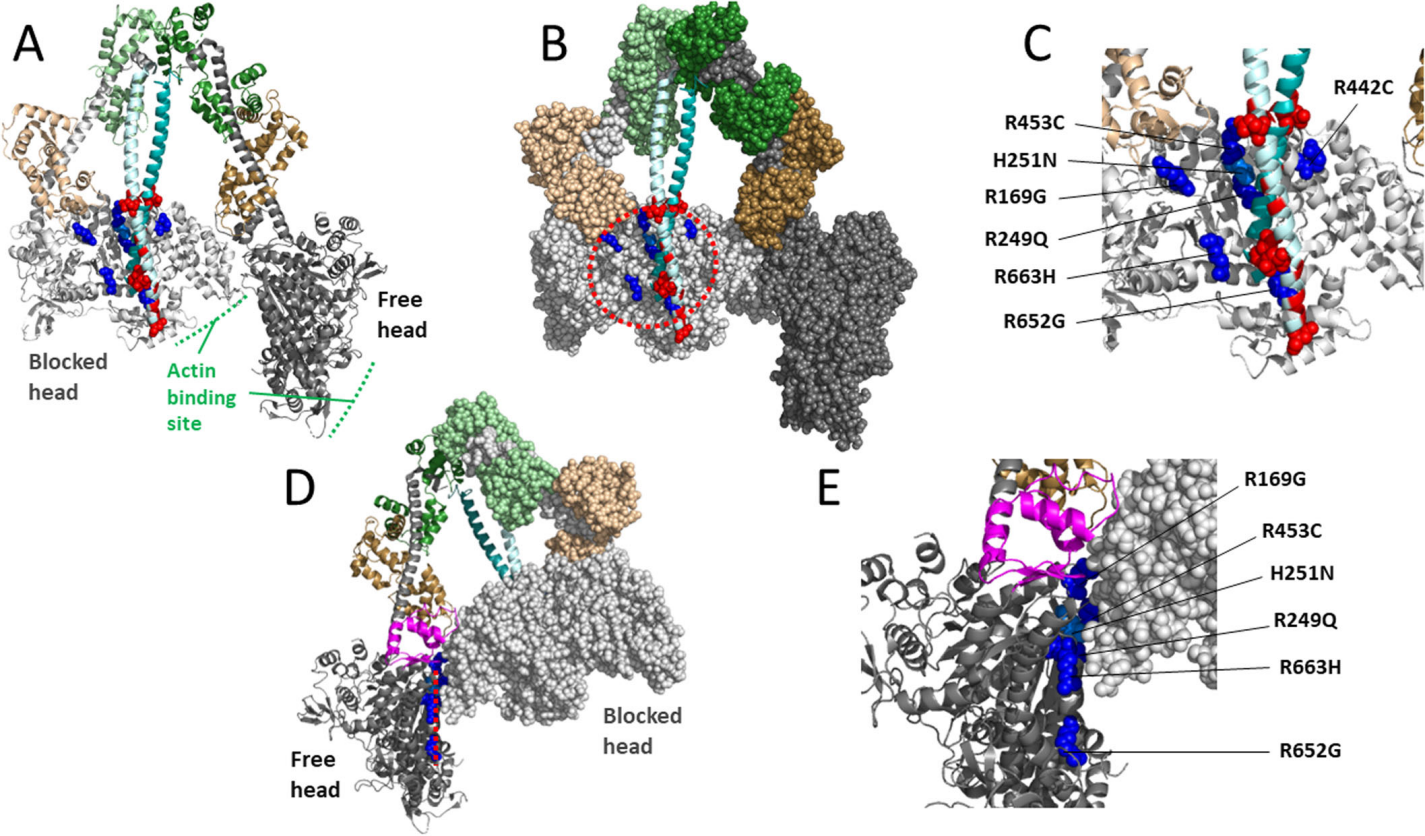
The 3.6 Å-resolution human β-cardiac myosin IHM structure (PDB 8ACT) with mesa residues at protein–protein interfaces. (**A**) Pymol cartoon configuration with the 6 arginine-based mesa HCM residues (R169G; R249Q; R442C; R453C; R652G; R663H) shown in dark blue Pymol sphere configuration and the single histidine-based HCM residue (H251N) shown in light blue (all on the blocked head only). The glutamate and aspartate residues in the proximal S2 near the mesa are colored red, with the four HCM residues (E875del; E894G; E903Q/K; D906G) shown as spheres. The blocked head myosin heavy chain is in light gray, its essential light chain (ELC) in light brown, and its regulatory light chain (RLC) in light green. The free head myosin heavy chain is in dark gray, its ELC in dark brown, and its RLC in dark green. The coiled-coil proximal S2 tail domain is shown in light and dark cyan. (**B**) The same configuration as in panel (**A**), but with both S1 heads shown as spheres. The dashed red oval outlines the blocked head mesa. (**C**) Blowup of the blocked head mesa region of panel (**A**). (**D**) The molecule in (**B**) rotated ~180° and with only the blocked head shown as spheres. The free head mesa is indicated by the dashed line and the mesa arginine and histidine residues are shown in blue sphere configuration. The free head converter domain is shown in magenta. (**E**) Blowup of the head–head interaction site shown in panel (**D**).

The IHM is said to have a blocked head (its actin binding face is unable to bind to actin) and a free head (the actin binding domain is more accessible) (Fig. 2). Margaret Sunita, a postdoctoral fellow in the Spudich/Ruppel laboratory, created homology models of human β-cardiac myosin folded into the IHM state (e.g., MS03 (Nag et al, 2017), https://spudlab.stanford.edu/homology-models), and this model made it immediately apparent that the cluster of positively-charged arginine residues on the myosin mesa that cause HCM when mutated (Spudich, 2015; Homburger et al, 2016) are in the vicinity of a cluster of negatively-charged glutamate and aspartate residues in the myosin proximal tail, many of which also cause HCM when mutated. Two other homology models of human β-cardiac myosin IHM, Protein Data Bank (PDB) accession code 5TBY (Alamo et al, 2017) and PDB code MA1 (Robert-Paganin et al, 2018), similarly predicted that the major molecular consequence of several HCM mutations appeared to be disruption of IHM interactions stabilizing the sequestered state of myosin. Alamo et al, (2017) and Robert-Paganin et al, (2018) have discussed in great detail the proposed direct and indirect effects of over 100 pathogenic HCM variants on the IHM stability, and both models have also been used to predict the effects of several dilated cardiomyopathy (DCM) mutations on the IHM. Overall, the three models have been very useful for many studies, but they are only rough models that do not provide sufficient resolution to say anything about specific sidechain interactions. Therefore, we have emphasized that obtaining the actual human β-cardiac myosin IHM structure was a high priority, as it could be quite different from the homology models.

In 2023, a 3.6 Å resolution structure of the human β-cardiac myosin IHM was solved, which was indeed significantly different in many important ways from any of the earlier homology models, as described (Grinzato et al, 2023). All previous models were wrong in significant ways (as described in the Grinzato et al, 2023 paper). Three high-resolution cryo-EM structures of smooth muscle myosin IHM were reported not long before the human β-cardiac myosin IHM structure was determined (Scarff et al, 2020; Yang et al, 2020; Heissler et al, 2021), and it was suggested that homology modeling based on these structures might reveal the effects of HCM mutations on cardiac IHM (Scarff et al, 2020). But comparisons of smooth muscle myosin and cardiac myosin IHM structures revealed major differences between them (Grinzato et al, 2023), establishing that such smooth muscle IHM-based analyses would have been wrong too. The high-resolution structure of the isolated human β-cardiac myosin IHM is compatible with the IHM observed in relaxed filaments solved at medium to low-resolution by cryo-EM and cryo-ET published recently (Dutta et al, 2023; Tamborrini et al, 2023). However, the high-resolution structure (PDB 8ACT, (Grinzato et al, 2023)) is the only one to provide atomic details needed to establish which conformation the heads must adopt to form the asymmetric IHM configuration.

The new structure reveals the same clustering of positively-charged arginine residues on the myosin mesa of the blocked head of the IHM near the proximal S2 (Fig. 2A–C), as observed in the MS03 homology model (Nag et al, 2017), and it is likely that a general charge–charge interaction between this arginine-rich cluster and the negatively-charged nearby region of the proximal S2 (Fig. 2, red residues; those shown as spheres being residues that when mutated cause HCM) helps stabilize the IHM state. And as was proposed earlier (Spudich, 2015; Spudich et al, 2016; Alamo et al, 2017; Nag et al, 2017; Robert-Paganin et al, 2018; Trivedi et al, 2018; Spudich, 2019), single residue changes in either of these clusters might result in weakening the IHM state, which results in more myosin heads available for interaction with actin. In the recent paper on the high-resolution structure of the human β-cardiac myosin IHM, it was pointed out that only arginine 453 has specific contacts with the proximal S2 (Grinzato et al, 2023). One important perspective, however, is that the mesa-proximal S2 interaction might not be dominated by specific sidechain interactions, but rather a more diffuse yet overall strong electrostatic interaction formed by a cloud of negative charges on the proximal S2 and a cloud of positive charges on the blocked head. Thus, the proximal S2 domain shown in Fig. 2 might not be fixed in position as shown in this static structure but might be able to dynamically access the broader positively-charged mesa surface. This complementary electrostatic charge surface is likely a prerequisite to form the IHM and to allow a dynamic on/off switch of the heads either packed against the thick filament or available for participating in contraction. In fact, further investigation revealed that the S2 coiled-coil can adopt different positions on the surface of the blocked head when IHM are in solution (Houdusse lab and Spudich lab, unpublished results), in agreement with the role of these complementary long range and dynamic interactions. The same arguments might apply to the head–head interaction site. Interestingly, unlike the earlier MS03 homology model where the head–head interaction site on the free head side primarily involved the converter domain (Nag et al, 2017), the 3.6 Å resolution structure of the human β-cardiac myosin IHM shows that a good portion of the free head mesa domain is involved (Grinzato et al, 2023) (Fig. 2D,E). The HCM residues R169G, R453C, H251N, R249Q, and R663H are all near this interface. As reported earlier, in the static structure, interactions are seen only with R169, R453, and H251 (Grinzato et al, 2023). However, in the dynamic structure, the overall cluster of positive charge might play a role in the formation and stabilization of the IHM.

These dynamic and plastic charge–charge interactions are important for tuning the heads available for heart contraction because the IHM is poised to unfold its heads with just a single charge change, such as any of the HCM arginine residues shown in Fig. 2, by way of an overall net charge change. As discussed below, however, HCM mutations nearly anywhere in the molecule appear to weaken the IHM state. Thus, the very precise structure of the human β-cardiac myosin IHM appears to be required to maintain the normal wild-type level of IHM heads in a population of myosin molecules. If the cloud cluster concept dominated the stability of the IHM state, then one would expect that primarily those HCM residues at protein–protein interaction sites would cause an opening of heads, while HCM mutations elsewhere in the molecule might predominantly cause changes in one of the three fundamental parameters of ATPase, F_intrinsic_, and velocity. As described below, this is not the case.

## MyBP-C regulates the stability of the sequestered OFF-state

One sarcomeric protein that could stabilize the heads in an OFF state is myosin-binding protein C (MyBP-C). In support of this proposal (Spudich, 2015; Spudich et al, 2016; Trivedi et al, 2018; Spudich, 2019) mutations in the β-cardiac myosin and MyBP-C genes account for ~80% of all known HCM mutations. MyBP-C has been shown in multiple studies to inhibit myosin interaction with actin, potentially by sequestering heads away from the thin filament (Gruen and Gautel, 1999; Gruen et al, 1999; Ababou et al, 2007; Oakley et al, 2007; Ababou et al, 2008; Ratti et al, 2011; Pfuhl and Gautel, 2012; Previs et al, 2012; Kampourakis et al, 2014; Mun et al, 2014; Previs et al, 2014; Moss et al, 2015; Inchingolo et al, 2019; Rahmanseresht et al, 2021; Brunello and Fusi, 2024). Consistent with this idea, Nag, Trivedi et al, showed that MyBP-C binds to the single-headed human β-cardiac myosin subfragment 1 (S1) (Nag et al, 2017). Thus, HCM-causing mutations in MyBP-C that lead to haploinsufficiency and decreased levels of MyBP-C in the sarcomere would result in more heads available for actin interaction, resulting in hypercontractility. Furthermore, like mutations on the myosin mesa, point mutations in MyBP-C that cause HCM may also weaken the proposed MyBP-C/myosin head interaction. Measurements in muscle fibers from HCM patients indicate that mutations in *MyBPC3* decrease the level of SRX (super-relaxed state), a low energy state of myosin (Hooijman et al, 2011) associated with the IHM structural state. These results support a role of this protein in stabilizing the IHM (McNamara et al, 2017; Toepfer et al, 2019). Nelson et al, recently elegantly determined the sub-sarcomeric location of SRX myosin in isolated mouse cardiac myofibrils (Nelson et al, 2023). Determination of the location of individual fluorescent-ATP turnover events using super-resolution microscopy revealed the presence of SRX myosin in a gradient along the thick filament: highest in C and P zones and lower in the D zone which lacks MyBP-C and lies farthest from the sarcomere center, suggesting that MyBP-C possibly stabilizes SRX myosin. Interestingly, myofibrils from *MYBPC3* null mice displayed roughly 40% reduction in SRX myosin in both C and D zones, suggesting that the effect of MyBP-C on SRX stability somehow extends beyond the C-zone. Similarly, Pilagov et al, observed a gradient of SRX myosin heads in skeletal muscle myofibers using a similar experimental approach (Pilagov et al, 2022), supporting a role for MyBP-C in regulating the stability of SRX myosin. HCM mutations in both myosin and MyBP-C can weaken this regulatory interaction, which may alter the stability of SRX myosin. A few HCM mutations in *MYH7* (R403Q, R870H, E924K, and E930del) (Sarkar et al, 2020; Singh et al, 2021) and *MYBPC3* (R502W) (Sen-Martín et al, 2024) have indeed been shown to weaken the myosin-MyBP-C interaction.

**Figure 3:**
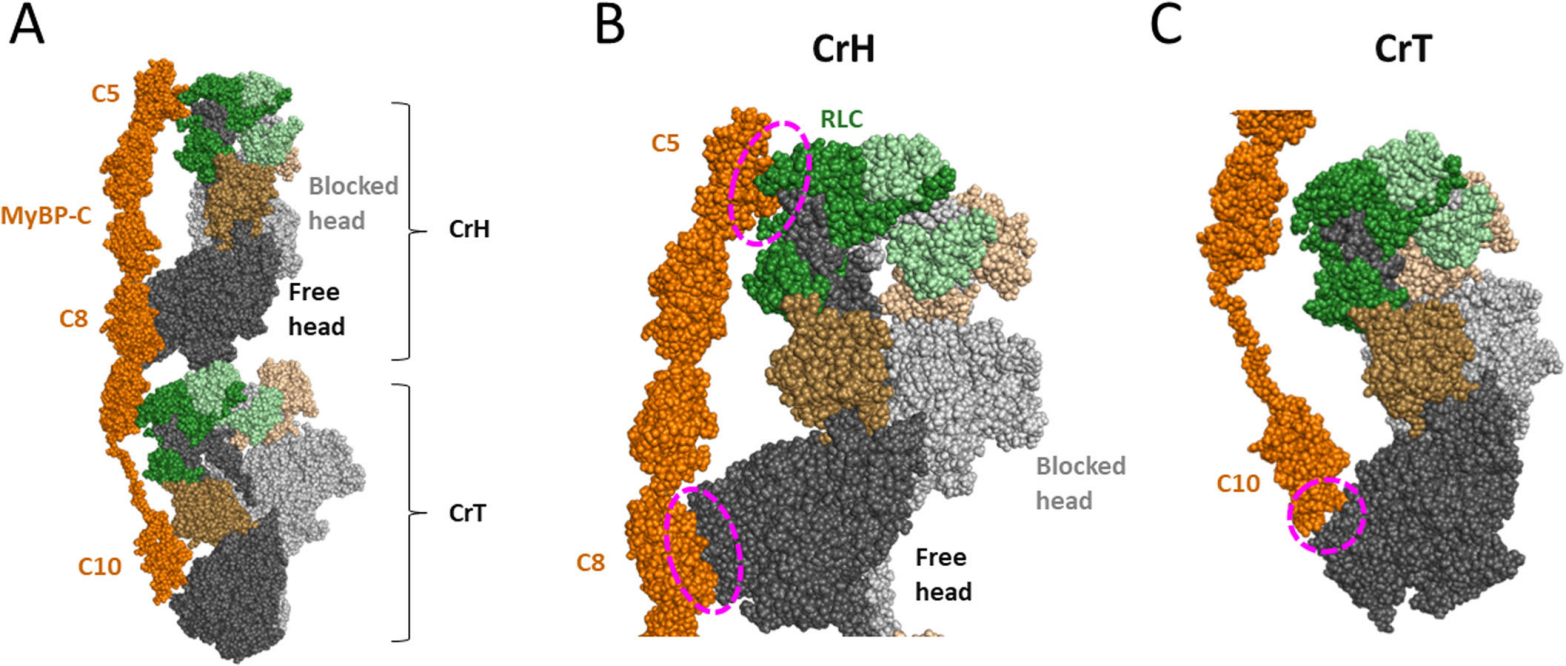
MyBP-C interacts with two IHM motifs in the relaxed cardiac filament. (**A**) View of the horizontal (CrH, Crown 1) and the tilted crown (CrT, Crown 3) from the structure of the cardiac filament in relaxed conditions (PDB code 8G4L). These two IHM motifs interact with the MyBP-C shown in orange. (**B**) Zoom on the CrH, showing the interactions of the free head with the C5 and C8 domains of MyBP-C. (**C**) Zoom on the CrT or Crown 3, showing the interactions of the free head U50 subdomain with the C10 domain of MyBP-C.

More biochemical measurements are needed to understand the role of myosin and MyBP-C in regulating contraction, but structural insights are critical to map the elusive myosin/MyBP-C binding interfaces. The recent cryo-EM and cryo-ET structures of the cardiac filament in relaxed conditions allow one to visualize for the first time the interactions between cardiac IHM and MyBP-C (Dutta et al, 2023; Tamborrini et al, 2023). In these structures, the cardiac filament has a typical three-fold pseudo-symmetry, and MyBP-C interacts with two of the IHM crowns: the horizontal crown (CrH, Crown 1) and the tilted crown (CrT, Crown 3) (Dutta et al, 2023; Tamborrini et al, 2023) (Fig. 3A). Three sites of interaction can be described from these structures. On Crown 1 (CrH), MyBP-C forms two regions of interaction with the free head: the C5 domain interacts with the RLC, and the C8 domain interacts with the U50 subdomain of the myosin head (Fig. 3B). The U50 domain of Crown 3 (CrT) interacts with the C10 domain of MyBP-C (Fig. 3C). These interactions support the hypothesis that the MyBP-C can stabilize the IHM. The mesa is mainly involved in the interfaces between the myosin heads and is found on the other side of the myosin/MyBP-C interfaces. Crown 2 (CrD) on the relaxed filament appears disordered and is far away from the MyBP-C C5-C10 domains.

The resolution of the current structures of the cardiac filament in relaxed conditions is not sufficient to describe the interactions between the IHMs and MyBP-C at an atomic level. Therefore, further investigations are critically needed to precisely define the role of MyBP-C on the dynamic formation/destabilization of the IHM motifs.

## Biochemical assays for measuring the percentage of myosin heads in the IHM state are essential

While structures are critical for understanding mechanisms of protein function, they can only lead to hypotheses, and biochemical assays are essential for examining, in this case, whether myosin HCM mutations liberate heads from an OFF state so there are more myosin heads accessible for interaction with actin. One biochemical assay that has been used to examine whether HCM mutations liberate myosin heads from an OFF state is the SRX assay already mentioned above, developed by Roger Cooke and his colleagues. These investigators made the important discovery that in relaxed fibers from both skeletal and cardiac muscle there exists a myosin state that has extremely low basal ATPase activity, meaning the level of myosin ATPase in the absence of actin interaction (Stewart et al, 2010; Cooke, 2011; Hooijman et al, 2011; Naber et al, 2011). The assay they developed involves loading fluorescently labeled ATP (mant-ATP) onto the myosin in permeabilized muscle fibers and measuring the rate of release of fluorescent nucleotide after chasing with excess unlabeled ATP. They called the population of heads having a very low basal ATPase rate the super-relaxed state (SRX). The population of heads with normal basal activity is called the disordered state (DRX) and their ATPase activity is quite low compared to the actin-activated ATPase rate, but the SRX rate is an order of magnitude lower than the normal basal rate, thus preserving energy utilization when the muscle is at rest. They estimated that about half of the myosin molecules in the relaxed state of the muscles are in the SRX state. In cardiac muscle, unlike skeletal muscle, these SRX heads remain even after the muscle is activated for contraction (Stewart et al, 2010; Hooijman et al, 2011; McNamara et al, 2015). Thus, in cardiac muscle, the SRX heads appear to be held in reserve and only activated when an increased power output of the heart is needed physiologically, for example by phosphorylation of the myosin RLC by myosin light chain kinase. Rohde et al, subsequently showed that the SRX state can be observed using purified bovine cardiac myosin (Rohde et al, 2018), and Anderson et al, showed the same with purified human β-cardiac myosin (Anderson et al, 2018), which has about 40% of its myosin heads in the SRX state, indicating that this state is linked to a conformation the myosin heads can adopt, rather than their sequestration by interactions with other components of the sarcomere.

This SRX assay has been used to estimate the fraction of heads in a presumed IHM OFF state in tissue samples of HCM patients and in iPSC-derived cardiomyocyte cell lines (Table 2). In 2017, McNamara et al, reported for the first time the presence of SRX myosin in human left ventricular (LV) tissue and showed that missense and truncation HCM mutations in *MYBPC3* disrupted SRX myosin using LV tissue samples obtained from HCM patients during myectomy surgery (McNamara et al, 2017). Similar observations of decreased SRX myosin proportion in human HCM heart fibers with the heterozygous *MYH7* mutation R663H (Anderson et al, 2018) or *MYBPC3* truncations (caused by frame-shift mutations: N981fs, L1014fs, and K1209fs) (Toepfer et al, 2019) have been made by other investigators. More recently, to better understand the early mechanisms of HCM disease manifestation due to mutations in *MYH7*, investigators from several laboratories have used cardiomyocytes derived from human induced pluripotent stem cells (hiPSC-CMs) CRISPR-edited to harbor a pathogenic HCM mutation in one of the *MYH7* alleles as model systems (Toepfer et al, 2020; Vander Roest et al, 2021; Lee et al, 2024). HCM mutations have been reported to evoke several key features of HCM pathophysiology including increased contractile function, altered cell size and myofibril organization, and altered cellular metabolism in these hiPSC-CM model systems. Three HCM mutations in *MYH7* studied using the iPSC-derived cardiomyocyte cell lines—R403Q, V606M, and R719W—were found to significantly decrease the proportion of myosins in the SRX state (Toepfer et al, 2020). Overall, in all cases examined, a decrease in SRX was observed, consistent with the hypothesis that HCM mutations cause a decrease in the number of heads held in a sequestered OFF-state, and this was true for HCM mutations in both MYH7 and *MYBPC3*, in keeping with the general unifying hypothesis.

**Table 2:**
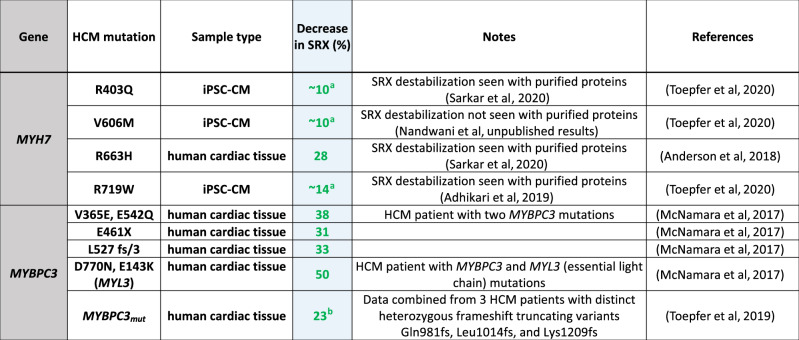
SRX measurements from tissue samples of HCM patients and in iPSC-derived cardiomyocyte cell lines.

Table showing percent decrease in SRX estimated from published studies of mant-ATP experiments from human HCM patient myocardial samples or iPSC-CMs carrying either *MYH7* or *MYBPC3* mutations. % decrease SRX = (WT−x)/WT, where x = the measured SRX level for a particular HCM mutant and wild-type (WT) control data from the same study was used as reference. The green values are generally assumed to indicate more myosin heads in play leading to hypercontractility (^a^estimated from Fig. 2F in Toepfer et al (2020), ^b^estimated from Fig. 3F in Toepfer et al (2019)).

Importantly, a similar level of SRX (40–50%) as seen in muscle fibers is also observed in purified human β-cardiac myosin preparations fully reconstituted with human β-cardiac myosin light chains (Adhikari et al, 2019; Sarkar et al, 2020; Vander Roest et al, 2021; Morck et al, 2022; Lee et al, 2024; Nandwani et al, 2024) (Table 1). Notably, as for the tissue samples of HCM patients and iPSC-derived cardiomyocytes, an HCM-induced increase in SRX has not been seen in these purified protein studies. All results show a decrease in SRX, consistent with the assumed increase in the number of heads accessible for interaction with actin caused by HCM mutations. Interestingly, the opposite was recently reported for two dilated cardiomyopathy (DCM) associated mutations, E525K in the heavy chain (Rasicci et al, 2022; Duno-Miranda et al, 2024) and D94A in the RLC (Yuan et al, 2022), both of which show an increase in SRX, and the assumed decrease in the number of myosin heads available for force production is proposed to be the primary mechanism of hypocontractility associated with these mutations. A logical assumption has been that the biochemical SRX state can be equated with the structural IHM state. However, the situation is more complicated than this, as described below, and caution needs to be exercised in using this indirect assay.

A few assays probing the IHM structure in solution have been developed to study the correlation between the biochemical SRX state and the structural IHM state. Fluorescence resonance energy transfer (FRET) based approaches have been designed where an increase in FRET efficiency is expected when the head–head (RLC-RLC) (Chu et al, 2021) or head-tail (Rasicci et al, 2022) interfaces of the IHM form, which brings the donor and acceptor fluorophores closer together. Interestingly, while the FRET sensor developed by Rasicci et al, showed a strong correlation between the SRX and IHM state as a function of ionic strength and the SRX-stabilizing E525K DCM mutation (Rasicci et al, 2022), the sensor developed by Chu et al, found uncoupling between the IHM and SRX (Chu et al, 2021). The insights derived from FRET depend critically on the spectral properties of the donor and acceptor fluorophores used as well as their placement. The two sensors described in the above work were not developed based on the high-resolution structure of β-cardiac myosin IHM (Grinzato et al, 2023), which could be one of the reasons why the results from the two FRET approaches seem to disagree. In fact, a FRET sensor developed in our lab based on the high-resolution structure of the cardiac IHM (PDB ID: 8ACT) shows a strong correlation between the SRX and IHM state as a function of ionic strength, proximal S2 tail length and cardiomyopathy mutations (Goluguri et al, unpublished results).

Another approach is negative stain electron microscopy (EM) which allows the configuration of myosin heads to be directly visualized. Using EM, stabilization of the SRX state by mavacamten (Anderson et al, 2018), a small molecule myosin inhibitor now approved for treatment of obstructive HCM, and the E525K DCM mutation (Rasicci et al, 2022) correlates with an increase in the number of molecules with folded-back (closed) heads. However, EM is not very quantitative because of the known disruptive effect of the EM grid on IHM stability.

**Figure 4:**
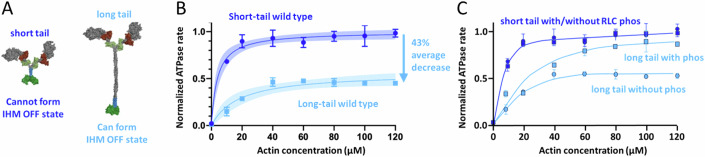
The long-tail/short-tail ATPase ratio (LSAR) assay. (**A**) Left, Pymol depiction of a 2-headed human β-cardiac myosin construct with a 2-heptad repeat of its proximal S2 stabilized by a GCN4 insert (blue), with a GFP (green) added to its C-terminus (short-tail construct). Right, Pymol depiction of a two-headed human β-cardiac myosin construct with a 25-heptad repeat of its S2 stabilized by a GCN4 insert, with a GFP added at its C-terminus (long-tail construct). (**B**) Actin-activated ATPase of a wild-type short-tail construct (dark blue) compared to a wild-type long-tail construct (light blue). Data from (Adhikari et al, 2019). (**C**) Actin-activated ATPase of a wild-type short-tail construct without (circles, dark blue) and with (squares, dark blue) RLC phosphorylation compared to a wild-type long-tail construct, without (circles, light blue) and with (squares, light blue) RLC phosphorylation. Data from (Nag et al, 2017). Data points in panels (**B**) and (**C**) are mean and s.e.m. from *n* = 2 independent protein preparations with 2–3 experimental replicates per preparation.

A direct biochemical assay for measuring whether an HCM mutation causes the release of more myosin heads for interaction with actin involves comparing the actin-activated ATPase activity of two different 2-headed human β-cardiac myosin constructs—a short-tail construct (2-heptad repeat, or more recently 8-heptad), which cannot form the IHM state, with the actin-activated ATPase activity of a long-tail construct (25-heptad repeat, or more recently a 15-heptad proved to be sufficient), which can form the IHM state (Nag et al, 2017; Anderson et al, 2018; Grinzato et al, 2023; Nandwani et al, 2024) (Fig. 4A). The actin-activated ATPase activity of a long-tail construct is typically 40–50% lower than its corresponding short-tailed construct (Fig. 4B), presumably because the long-tail construct can form the IHM OFF state. Of more than 100 measurements over several years assessing the ratio of actin-activated ATPase for the long-tail myosin wild-type construct and the short-tail myosin wild-type construct, the value has consistently been between 0.5 and 0.6, with the average value being 0.57. This is presumably due to 43% of the population of wild-type human β-cardiac myosin existing in an IHM state. If an HCM mutation destabilizes the putative IHM state in these biochemical measurements, then the long-tail constructs will approach the ATPase values of the short-tail constructs. We call this the long-tail/short-tail ATPase ratio (LSAR) assay.

Another assay that biochemically assesses the effect of myosin’s autoinhibition on interaction with actin was recently developed by the Warshaw laboratory (Duno-Miranda et al, 2024). These authors used the observation that a myosin-concentration dependence on velocity of actin filaments can be measured if methylcellulose is included to prevent actin filaments from diffusing away from a myosin-coated surface during motility measurements (Uyeda et al, 1990). In this assay, myosin density-dependent in vitro motility experiments were performed using 2-headed short and long-tailed myosin constructs, and an analytical model was developed to predict the fraction of heads in the IHM/SRX conformation from these measurements. This assay provides a readout of how myosin autoinhibition impacts motor-based actin sliding and thus can be correlated with contractility in muscle.

## The effects of physiologically relevant biochemical changes and HCM mutations on opening of myosin heads can be examined by the LSAR assay

Nag et al, used the LSAR assay to examine the effects of RLC phosphorylation on the number of OFF-state heads in a myosin population (Nag et al, 2017). In their experiments, a short-tail myosin construct showed the same level of actin-activated ATPase regardless of the RLC phosphorylation state. However, an unphosphorylated long-tail construct had reduced actin-activated ATPase, presumably because a significant percentage of the myosin heads in the non-phosphorylated state are in an IHM state, and phosphorylation of the RLC of the long-tail myosin largely eliminates the OFF-state population (Fig. 4C). This directly demonstrates biochemically the likely physiological role of RLC phosphorylation in cardiac muscle, as described for multiple myosin types previously (Craig et al, 1987; Padrón et al, 1991; Cremo et al, 1995; Levine et al, 1996; Trybus et al, 1997; Wendt et al, 1999; Alamo et al, 2008; Lowey and Trybus, 2010; Scruggs and Solaro, 2011; Toepfer et al, 2013; Alamo et al, 2015; Espinoza-Fonseca et al, 2015; Kampourakis and Irving, 2015; Vandenboom, 2016; Trivedi et al, 2018).

When using the LSAR assay to measure whether HCM mutations cause an increase in the LSAR, which indicates the opening of heads from a sequestered state, all experiments have used constructs with the RLC in its non-phosphorylated state. Furthermore, for every mutation, both the mutant short-tail version and the mutant long-tail version were made and the actin-activated ATPase of the two were compared. This comparison is essential since some HCM mutations result in a change in the fundamental actin-activated ATPase rate parameter of the short tail version (Table 1, first blue column), also seen in single-headed S1 experiments (Nag et al, 2015; Adhikari et al, 2016; Vera et al, 2019; Morck et al, 2022; Nandwani et al, 2024).

Twenty HCM mutations have now been analyzed using both the LSAR and SRX assays (Table 1, Fig. 1D), and there are several important conclusions. First and foremost, 19 of the 20 total show an increase in the number of myosin heads in the ON state using the LSAR assay, presumably by weakening the IHM configuration. The single mutation that did not show an increase in heads in an ON state using the LSAR assay, I457T, demonstrated a large 75% increase in actin-activated ATPase and a 240% increase in velocity (Table 1, blue columns) (Adhikari et al, 2019). These values significantly exceed the changes in these fundamental parameters observed for any of the other mutations, and presumably account for the hypercontractility caused by the I457T mutation. The bottom-line, however, is that it is likely that most HCM mutations in myosin cause an increase in the number of heads available for interaction with actin (N_a_), in support of the unifying hypothesis proposed in 2015 (Spudich, 2015).

Table 1 shows that several HCM mutations affect intrinsic motor properties (k_cat_, velocity and intrinsic force) in significant ways, and these changes occur in both hypo and hypercontractile directions. The overall effect of a mutation on contractility is expected to be impacted by all these factors as well as changes in N_a_, and therefore it is important to measure all these parameters. We adopted a multiscale experimental approach to characterize the P710R HCM mutation, which decreased activity at the level of the fundamental parameters of the motor domain but increased force generation at the cell level (Vander Roest et al, 2021). A computational model used to integrate all the observed changes revealed that the increased availability of myosin heads is an essential driver of hypercontractility for this mutation. It will be interesting to see how disease severity is impacted by the integrated molecular effects of a mutation on both N_a_ and myosin’s fundamental biomechanical properties.

**Figure 5:**
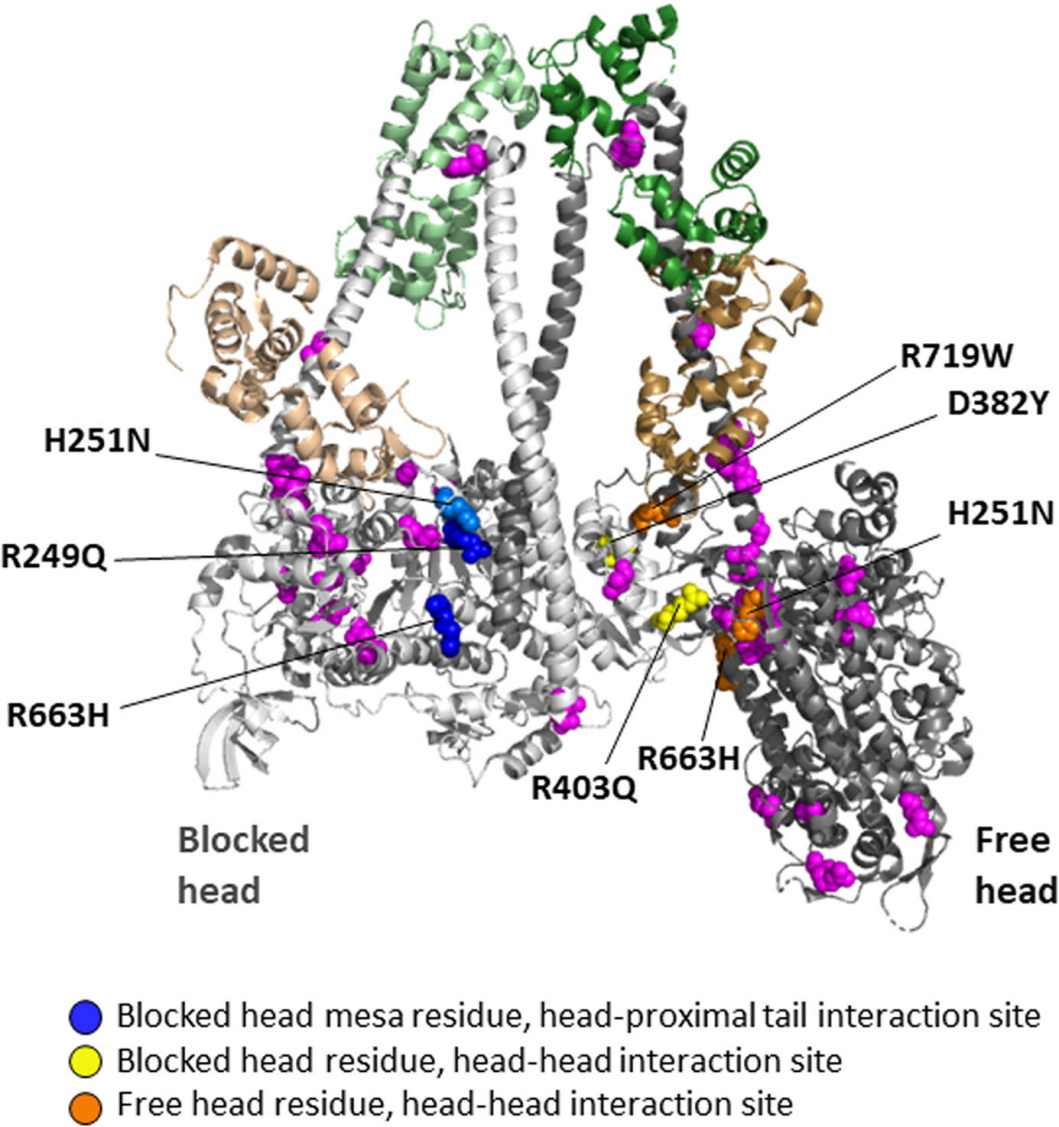
The PDB 8ACT IHM structure showing the locations of all residues for which LSAR data is available. The 6 out of the 20 HCM residues that have been analyzed using the LSAR assay that are in the vicinity of protein–protein interfaces of the IHM state are colored blue (on the blocked head at the head-proximal S2 interaction site), yellow (on the blocked head at the head–head interaction site), and orange (on the free head at the head–head interaction site). The remaining 14 analyzed HCM residues, which are clearly not at the protein–protein interaction sites, are colored magenta.

From a structural standpoint, 6 out of the 20 HCM residues that have been analyzed using the LSAR assay are in the vicinity of protein–protein interfaces of the IHM state (Fig. 5), R249Q on the blocked head at the head-proximal S2 interaction site (blue residue), D382Y and R403Q on the blocked head at the head–head interaction site (yellow residues), R719W on the free head at the head–head interaction site (orange residue), and H251N and R663H, which are near both the blocked head-proximal S2 interaction site and the head–head interaction site. Not all of these 6 are necessarily playing a role at the relevant interfaces (Grinzato et al, 2023), but the remaining 14 analyzed HCM residues (Fig. 5, magenta residues) are clearly not at the protein–protein interaction sites. Yet, 13 of them cause an opening of the heads from the IHM OFF state (Table 1). This is highly significant because those mutations must be altering the detailed atomic-resolution features of the molecule rather than causing a change in a surface charge cluster described above. This is why a high-resolution structure of the human β-cardiac myosin IHM was essential, since it allows one to study the interfaces stabilizing the motif at an atomic level and to describe the precise effect of each HCM-causing mutation.

The fact that many of the HCM mutations that cause an increase in N_a_ do not lie at obvious interfaces between the two myosin heads or the interface between the blocked head and the proximal S2 (see Table 1 and Fig. 5) emphasizes that one cannot predict from the structure alone whether a particular mutation is likely to increase N_a_. This is because myosin is a highly dynamic and allosteric protein and mutations anywhere can propagate changes to the other parts of the protein and influence the stability of the IHM state. Thus, it is essential to carry out biochemical assays to determine whether the effects of a particular mutation increase N_a_.

The second major observation from the data in Table 1 is that while 13 of these HCM mutations also show a decrease in the number of SRX heads, 7 of them do not. This emphasizes that while the SRX assay appears to be reasonably good at predicting an opening up of myosin heads, it must not be used as a definitive assay to establish whether molecules in the IHM OFF state are being liberated for interaction with actin because of the mutation. This caution goes along with the results of Anderson, Trivedi et al, which showed that both a short tail version of human β-cardiac HMM and single-headed S1 show a significant amount of SRX activity (Anderson et al, 2018). This was also observed for bovine cardiac S1 by Rohde et al, (2018). Similar disconnects were observed when SRX was correlated with IHM measured by FRET (Chu et al, 2021) or by the quasi-helical ordered arrangement of heads observed by X-ray diffraction (Ma et al, 2023; Jani et al, 2024). These results indicate that an IHM state is not needed for cardiac myosin to be in an SRX state, and IHM and SRX must not be equated (Anderson et al, 2018; Craig and Padrón, 2022).

Another important but less widely discussed issue relates to the kinetics of transition into and out of the SRX state. In myofibril measurements, entry into the SRX state was observed to be fast (>10 s^−1^) (Walklate et al, 2022) while the exit must be slower than the rate at which SRX myosin turns over ATP (0.002 s^−1^) for it to stably exist for hundreds of seconds as observed in the single-turnover assays (Walklate et al, 2022). The equilibrium constant with these rates of formation and decay of SRX does not agree with the equilibrium populations of DRX and SRX myosin typically observed in the single-turnover assays. These paradoxes have complicated the interpretation of the SRX state. Recently, some have even questioned whether distinct SRX and DRX populations can be observed using purified proteins in the mant-ATP single turnover assay (Mohran et al, 2024). More experiments are needed to interpret these findings because many of the studies using the single turnover assay are backed up by steady-state ATPase (LSAR), ATPase without actin, EM, FRET, and motility measurements that often agree with the number of active heads suggested from single turnover experiments (Anderson et al, 2018; Rohde et al, 2018; Adhikari et al, 2019; Sarkar et al, 2020; Vander Roest et al, 2021; Rasicci et al, 2022; Duno-Miranda et al, 2024; Lee et al, 2024; Nandwani et al, 2024). In any case, the LSAR assay is a reliable and direct biochemical assay for measuring whether an HCM mutation causes more heads to become available for actin interaction.

It is important to note the limitations of the LSAR assay. This assay cannot determine the fraction of IHM heads in the absence of actin, and it assumes that the number of IHM heads will be stable, even in the presence of actin. Previous data supporting a model where myosin autoinhibition can be influenced by actin (Rohde et al, 2018) indicates that this may not always be true. Furthermore, the LSAR assay must be performed at low ionic strength to achieve saturation of the actin-activated ATPase activity, an issue that has been true for the standard actin-activated ATPase assay since its inception. The IHM is sensitive to salt, and while for HCM mutations the difference from WT is expected to be most obvious at low salt where IHM is most stable, the effects of some mutations like the E525K DCM mutation which stabilizes the IHM may only be evident at high salt (Rasicci et al, 2022).

## Two small molecule inhibitors of human β-cardic myosin have been developed to reverse the effects of HCM-induced hypercontractility

While conventional therapies for HCM are aimed toward controlling the hyperactive physiology, until recently none have been directed toward inhibiting β-cardiac myosin directly. Over the last decade, however, two small molecule inhibitors that bind directly to the β-cardiac myosin head domain and inhibit its actin-activated ATPase activity have been described, mavacamten (Green et al, 2016) and aficamten (Chuang et al, 2021). Both molecules were developed with the concept that if one could reduce the hypercontractility to normal this could reduce the negative effects of the hypercontractile state and possibly prevent or even reduce existing hypertrophy of the heart (Green et al, 2016; Heitner et al, 2019; Spudich, 2019; Hegde et al, 2021; Saberi et al, 2021; Day et al, 2022; Kawana et al, 2022; Lehman et al, 2022; Maron et al, 2023).

**Figure 6:**
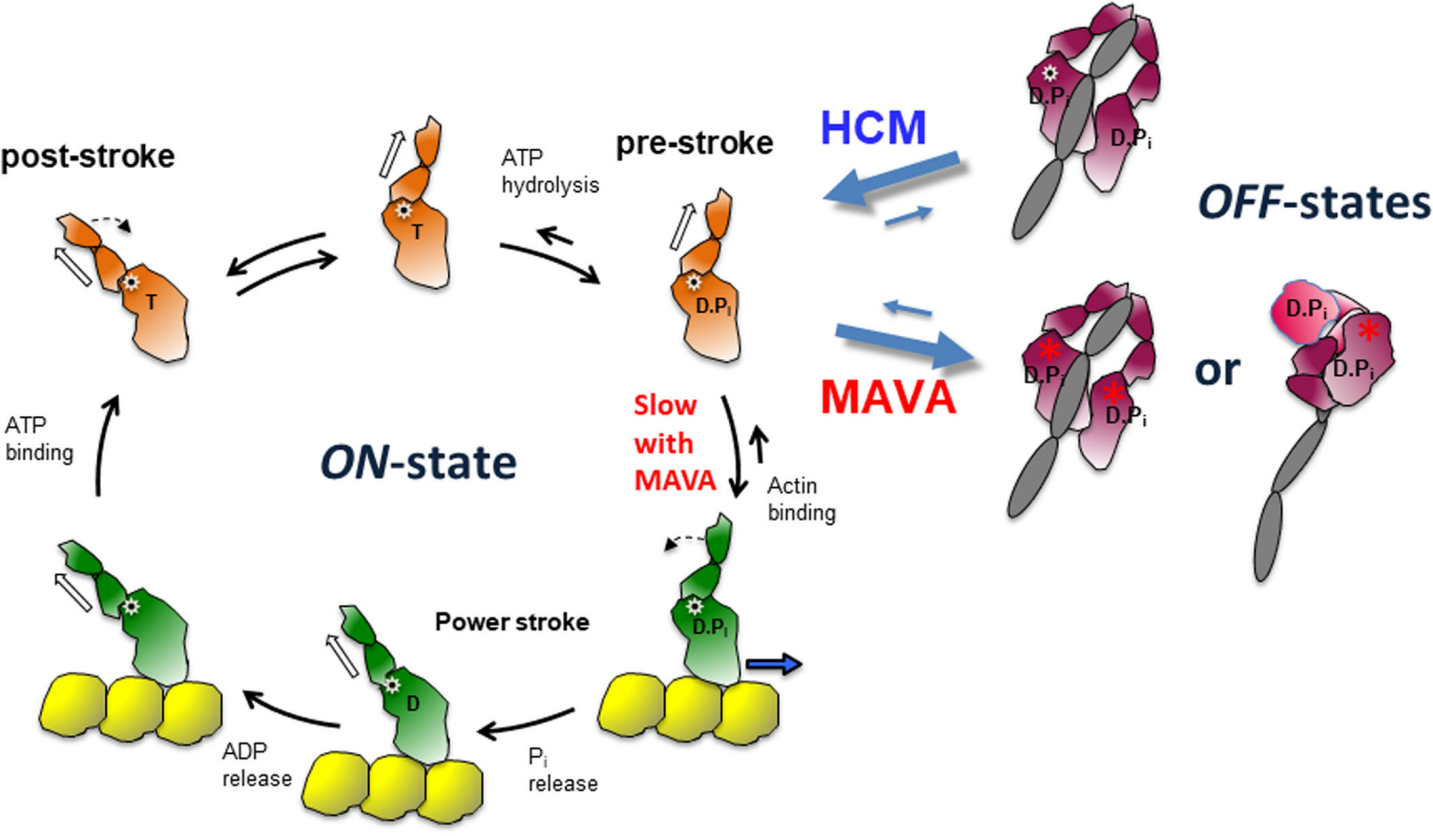
Schematic view of the actin-activated myosin ATPase cycle (left) and various forms of OFF states (right). The formation of the OFF-states removes myosin heads from the cycle. The red star indicates mavacamten binding.

Mavacamten reverses the primary action of HCM mutations by putting myosin heads back into a 2-headed-compact sequestered OFF state, as possibly suggested by EM (Anderson et al, 2018) and small angle X-ray scattering studies (Gollapudi et al, 2021). It is not clear whether mavacamten binding puts myosin heads back into an IHM state or into some other 2-headed-compact state (Fig. 6) (Chu et al, 2021; Gollapudi et al, 2021; Nag et al, 2023). In the cryo-EM and cryo-ET studies where the thick filament is relaxed by mavacamten (Dutta et al, 2023; Tamborrini et al, 2023), the structure of the IHM seems similar to that of apo classical IHMs (Grinzato et al, 2023). Thus, it will be essential to determine the high-resolution structure of the mavacamten-bound two-headed myosin to depict how the drug influences the conformation and dynamics of the IHM. Interestingly, molecular dynamics based on the crystal structure of cardiac myosin complexed to mavacamten demonstrated that mavacamten explores different positions in its allosteric binding pocket, which influences the dynamics of the lever arm as well as the allostery within the myosin head (Auguin et al, 2024). It is thus likely that the conformational space explored by the IHM in presence of mavacamten differs. In addition, mavacamten also acts by inhibiting the ability of a free head to produce force on F-actin (Auguin et al, 2024). In sum, mavacamten fundamentally reverses the effects of the HCM mutations, returning active heads back to a sequestered OFF state or slowing their ability to bind and produce force on F-actin.

## Conclusion

The results of the last nine years have led to considerable evidence in support of the unifying hypothesis that the key mechanism of HCM-induced hypercontractility is the release of myosin heads from a sequestered OFF state, putting more heads in play for interaction with actin. The OFF state has now been depicted at high resolution, revealing the interactions that stabilize the asymmetric IHM state (Grinzato et al, 2023), which is further sequestered on the thick filament by interactions with MyBP-C (Dutta et al, 2023; Tamborrini et al, 2023). We suggest that this unifying hypothesis not only pertains to HCM mutations in myosin but to HCM mutations in other sarcomeric proteins as well. As discussed above, the majority of HCM-causing mutations in *MYBPC3* lead to haploinsufficiency and therefore less MyBP-C to sequester myosin heads.

**Figure 7:**
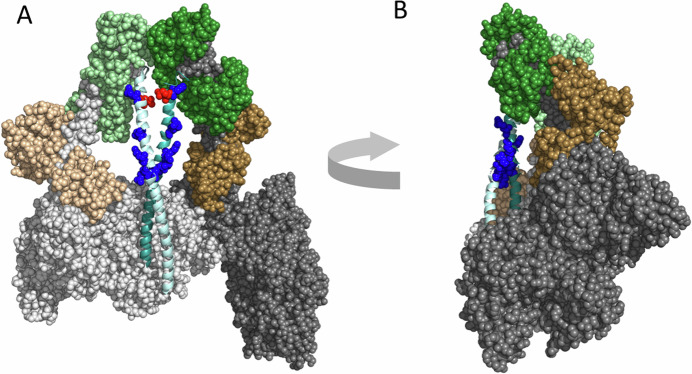
Mutations in the early region of proximal S2. (**A**) The 3.6 Å-resolution human β-cardiac myosin IHM structure (PDB 8ACT) with HCM mutations affecting charged residues in the first 4.5 heptads of the proximal S2 shown as blue spheres. (**B**) Image in (**A**) rotated ~90°. The mutations are K847E; E848G; R858C, P, S; K865E; R869H; R870H.

While the LSAR assay is an excellent direct assay for measuring changes in N_a_ for myosin HCM mutations, so far it has been applied only in the context of purified human β-cardiac myosin alone. Many of the myosin HCM mutations might require additional components for the effects on N_a_ to be seen. In the context of the thick filament, the heads of one myosin molecule interact with the heads of an adjacent molecule, and some HCM mutations in the myosin head may affect this stabilizing interaction, causing a weakening of the OFF-state (Grinzato et al, 2023; Dutta et al, 2024). In particular, HCM mutations in the myosin rod might involve residues that are involved in or interact with the light-meromyosin (LMM) core of the thick filament or interact with other protein components of the thick filament. In the proximal S2, for example, there is a hot spot for HCM mutations in the first 4.5 heptads of the rod, many of which change positively charged arginine and lysine residues to negatively charged or uncharged residues (Fig. 7). This initial region of the coiled-coil does not appear to be involved in stabilizing the IHM configuration as a purified protein according to the recent high-resolution WT structure (Fig. 7), and it is also ~4–5 nm away from the LMM core or other proteins currently modeled from the thick filament maps in the relaxed state (Dutta et al, 2023; Tamborrini et al, 2023). While it cannot be excluded that this cluster of positive residues might interact by weak charge–charge interaction with a net negative region of another protein of the thick filament to stabilize the OFF-state, it is likely that the mutations affect the dynamics/stability of the coiled-coil itself, thus leading to destabilization of the OFF-state configuration. Indeed, these residues are involved in van der Waals and electrostatic interactions between the two strands of the proximal S2 according to our high-resolution structure (Grinzato et al, 2023). These possibilities can now be explored by characterization of mutant recombinant cardiac myosin with the LSAR, biophysical, and structural assays. Further elegant cryo-electron microscopy and tomography maps of cardiac thick filaments containing mutant myosins studied up to ~6 Å and ~18 Å resolution, respectively, as recently described for WT cardiac samples (Dutta et al, 2023; Tamborrini et al, 2023) should also be performed to get a holistic description of how distinct mutations affect the number of heads in the OFF-state on the thick filament in relaxed conditions. By combining our 3.6 Å IHM structure with these cardiac thick filament reconstructions, it is now possible to analyze how these and other myosin HCM mutations might be affecting the IHM OFF-state in the context of the thick filament.

Regarding the issue of using SRX as an assay for measuring an increase in N_a_, the data in Table 1 emphasizes that caution should be used in interpreting SRX data. On the positive side, an SRX increase has not been seen in any of the HCM mutant studies, which would have suggested an increase of heads in an OFF-state (Rasicci et al, 2022; Yuan et al, 2022; Duno-Miranda et al, 2024), and the decreases in SRX found have always been confirmed with the LSAR assay. But there are 7 cases where a clear increase in N_a_ is seen by the LSAR assay that is not reflected in the SRX assay. Thus, the direct LSAR assay is the preferred approach but has the limitation that it can only be applied to examination of purified proteins.

Finally, along with a better understanding of the mechanistic basis of HCM-induced hypercontractility has come new therapies for HCM that target the human β-cardiac myosin directly with small molecules that remove myosin heads from accessibility to actin interaction, thus normalizing the contractility of the heart.

## Future directions

Regulation of the autoinhibited OFF state of myosin now appears to be a key determinant of cardiac contractility and energy utilization. Pathogenic mutation-associated alterations in myosin’s conformational dynamics are beginning to be associated with myopathies other than HCM including DCM and atrial cardiomyopathy (mutations in *MYH6*; Kawana et al, unpublished results). In the past few years, alterations in the sequestered OFF state stability by DCM-causing mutations have been observed for two mutations in myosin heavy (E525K) and light chains (D94A in RLC); both mutations were found to result in stabilization of the SRX state (Rasicci et al, 2022; Yuan et al, 2022; Duno-Miranda et al, 2024), which is predicted to lead to hypocontractility due to reduced number of myosin heads available for force production. FRET-based assays, negative-staining EM analysis and comparison of the actin-activated ATPase rates and in vitro velocities for long and short-tailed constructs of E525K indicate that the hypocontractility associated with this DCM mutant can be attributed primarily to increased sequestration of myosin heads in the IHM/SRX state (Rasicci et al, 2022; Duno-Miranda et al, 2024). DCM, however, is more heterogenous than HCM and may lack a unifying mechanism of disease manifestation, as indicated by results from other studies where pathogenic mutations including the novel severe early-onset DCM mutation Q222H (Kawana et al, 2023) and R369Q (Nandwani et al, unpublished results) were investigated and were found to not affect the SRX-DRX equilibrium.

Recent studies have implicated the destabilization of the OFF state of *MYH7* in the pathogenesis of skeletal myopathy as well. Using muscle biopsy specimens obtained from patients, molecular analysis of 10 distinct *MYH7* mutations in the light-meromyosin (LMM) core of the thick filament, which result in skeletal muscle diseases rather than cardiomyopathies, found a significant decrease in the proportion of SRX myosin (Carrington et al, 2023; Buvoli et al, 2024). It remains to be seen how altered SRX-DRX equilibrium contributes to the pathophysiology of skeletal muscle myopathies.
